# Effect of Postoperative Oral Intake Status on Sarcopenia Six Months After Esophageal Cancer Surgery

**DOI:** 10.1007/s00455-022-10471-z

**Published:** 2022-06-18

**Authors:** Nanako Hijikata, Aiko Ishikawa, Satoru Matsuda, Michiyuki Kawakami, Kaori Muraoka, Makiko Ando, Shuhei Mayanagi, Tomoyuki Irino, Hirofumi Kawakubo, Yuko Kitagawa, Tetsuya Tsuji

**Affiliations:** 1 Department of Rehabilitation Medicine, Keio University School of Medicine, 35 Shinanomachi, Shinjuku-ku, Tokyo, 160-8582 Japan; 2 Department of Rehabilitation Medicine, National Cancer Center Hospital East, Kashiwa, Chiba, Japan; 3 Department of Surgery, Keio University School of Medicine, Tokyo, Japan; 4 Department of Rehabilitation Medicine, Kitasato University Kitasato Institute Hospital, Tokyo, Japan; 5 Department of Rehabilitation Medicine, Keio University Hospital, Tokyo, Japan; 6 Division of Esophageal Surgery, Shizuoka Cancer Center Hospital, Shizuoka, Japan

**Keywords:** Dysphagia, Esophagectomy, Esophageal cancer, Oral intake, Sarcopenia, Videofluoroscopy

## Abstract

**Purpose:**

In patients with esophageal cancer, skeletal muscle mass has been reported to decrease progressively after surgery and be independently associated with a poor prognosis. The purpose of this study was to investigate perioperative changes in dysphagia, oral intake status, and nutritional status and identify factors related to sarcopenia 6 months after esophagectomy.

**Methods:**

A total of 134 patients who underwent radical resection for thoracic esophageal cancer between March 2016 and July 2019 were analyzed retrospectively. The diagnosis of sarcopenia was made by CT taken 6 months postoperatively using the cut-off criteria of skeletal muscle index (SMI) < 52.4 cm^2^/m^2^ for male and SMI < 38.5 cm^2^/m^2^ for female patients. As factors related to postoperative sarcopenia, dysphagia, oral intake status, nutritional status, and physical function were extracted from the medical records. Multivariate logistic regression analysis was performed to identify perioperative risk factors related to sarcopenia 6 months after surgery.

**Results:**

Of the 134 patients, 34.3% were judged to be unable to start oral intake on swallowing assessment. At discharge, 30.6% received tube feeding with or without oral intake. In the non-oral intake group on swallowing assessment, a significantly higher proportion of patients received tube feeding at discharge (*p* = 0.014). Preoperative BMI, postoperative handgrip strength, and tube feeding at discharge were independent risk factors for sarcopenia 6 months after esophagectomy in male patients.

**Conclusion:**

Tube feeding at discharge is significantly related to postoperative sarcopenia in patients with esophageal cancer. Identifying high-risk groups might allow early detection of malnutrition and provision of appropriate care.

**Supplementary Information:**

The online version contains supplementary material available at 10.1007/s00455-022-10471-z.

## Background

Sarcopenia is a syndrome characterized by decreased skeletal muscle mass, muscle strength, and physical function with advanced age [1]. Age-related changes, such as decreased anabolic hormones, increased inflammatory cytokines, and impaired signaling pathways in muscle synthesis, are primary causes of sarcopenia [2]. Sarcopenia is classified as primary with no other cause evident except aging and secondary with other causes evident, such as physical inactivity, systemic disorders, and malnutrition. Malignancy is one of the leading diseases causing secondary sarcopenia [3]. Patients with cancer are at high risk of malnutrition and changes in body composition including skeletal muscle mass as a result of anorexia, inadequate food intake, and abnormal metabolism [4]. Loss of skeletal muscle mass in cancer occurs by cachexia-associated mechanisms, in which cytokine-related systemic inflammation and oxidative stress arising from complex host-tumor interactions contribute to muscle and fat wasting [5].

For the assessment of sarcopenia, handgrip strength, the five-time chair stand test, or other methods measuring muscle strength are recommended to identify probable sarcopenia, and dual-energy X-ray absorptiometry (DXA), bioelectrical impedance analysis (BIA), magnetic resonance imaging (MRI), or computed tomography (CT) is recommended to confirm low muscle mass [6, 7]. In patients with cancer, CT is performed routinely during the oncological evaluation and is therefore considered a preferred method for assessing skeletal muscle mass in this population [8]. In particular, cross-sectional muscle area at the third lumbar vertebra best reflects total skeletal muscle mass [9] and can be used as the skeletal mass index (SMI), a normalized value for stature since the area is divided by the square of the height.

In patients with esophageal cancer, skeletal muscle mass has been assessed to investigate whether the presence of preoperative sarcopenia impacts short- and long-term outcomes. A recent review of esophageal cancer showed that preoperative sarcopenia is a significant risk factor for postoperative complications, including respiratory complications and anastomotic leakage, and survival [10, 11]. A recent study reported that skeletal muscle mass decreases progressively after esophagectomy, with the prevalence of sarcopenia increasing [12]. Postoperative loss of skeletal muscle has also been reported to be independently associated with a poor prognosis [13–15]. Patients with esophageal cancer often suffer from dysphagia preoperatively due to tumor-induced esophageal narrowing and are therefore prone to malnutrition. Neoadjuvant chemotherapy has been reported to reduce skeletal muscle mass due to impaired muscle cell proliferation and decreased protein synthesis [16]. After esophagectomy, it leads to continued gastrointestinal symptoms and dysphagia as a result of anatomical and functional changes [16]. Progressive weight loss follows postoperatively, with the greatest loss within 6 months of surgery, and it continues even after five years [17, 18]. Patients who were over 6 months after esophagectomy showed significantly lower skeletal muscle mass and worse physical functioning compared with age and sex-matched healthy controls [19]. Postoperative dysphagia, oral intake status, and nutritional status before and after esophagectomy might be related to postoperative skeletal muscle loss; however, studies investigating factors determining postoperative sarcopenia are lacking. To clarify perioperative factors associated with postoperative sarcopenia would be helpful for early detection of high-risk patients and provision of focused care, thus potentially improving the patients’ outcomes.

The purpose of this study was to investigate the changes from the preoperative period to 6 months after esophagectomy in dysphagia, oral intake status, and nutritional status as factors affecting postoperative skeletal muscle loss, and to identify perioperative factors determining sarcopenia 6 months after esophagectomy.

## Methods

### Study Design

This was a retrospective, observational study using the medical records of Keio University Hospital, Tokyo, Japan. In the present study, all patients who underwent one-stage radical resection for thoracic esophageal cancer between March 2016 and July 2019 at the Department of Surgery were included. CT was routinely performed 6 months after esophagectomy for follow-up, and patients in whom CT images were not available or were not taken by December 31, 2019 were excluded. This study was approved by the institutional ethics review board (20190268). The outline of the study was published on the institution’s public website, and the participants were guaranteed the right to refuse participation.

### Clinical Treatment and Perioperative Rehabilitation

Participants underwent esophagectomy with or without neoadjuvant therapy. Detailed surgical procedures and indications for neoadjuvant therapy have been presented elsewhere [20–22]. All patients hospitalized for esophagectomy were referred to the Department of Rehabilitation and received perioperative rehabilitation. Physical therapy consisted of preoperative pulmonary rehabilitation, postoperative early mobilization, walking training, and strengthening and endurance exercises. At the time of discharge, physical therapists provided instructions on home exercise programs including stretching, strengthening, and aerobic exercises. The content of speech therapy is described below.

### Dysphagia Rehabilitation and Initiation of Enteral Feeding and Oral Intake

Speech therapy consisted of preoperative clinical swallowing assessment and postoperative non-swallowing exercises started after extubation or tracheostomy. Enteral nutrition was started early after esophagectomy by a placed jejunostomy. Anastomotic leakage was assessed using CT and esophagography at postoperative day (POD) 7. Then, the patients without anastomotic leakage received swallowing assessment using a videofluoroscopic swallowing study (VFSS) and fiberoptic endoscopic evaluation of swallowing (FEES) at POD 8 or POD 12. VFSS consisted of 3 and 5 ml of thin and thickened liquids with iopamidol. Patients were seated upright or semi-reclined at a 60-degree angle and viewed in the lateral position. Aspiration was defined as an entry of bolus below the vocal folds, equivalent to penetration-aspiration scale (PAS) 6–8, and penetration was defined as entry of bolus into the airway above/on contact with the vocal folds (PAS 2–5) [23]. A rehabilitation physician and a speech therapist reviewed the findings and determined by consensus whether to start oral intake.

The consistency of food was changed according to swallowing function, from pureed, to soft, to regular food [24], and this modification required a longer time with more severe dysphagia. Swallowing training was performed by a speech therapist using head rotation, chin-tuck, and supraglottic swallowing maneuvers. The jejunostomy was continued for all the patients at discharge. If oral intake was not sufficient to satisfy the patient’s nutritional needs, alternative nutrition via the jejunostomy was used as a complement. Nutrition education was provided by a dietitian at discharge. The jejunostomy was removed after discharge when sufficient nutrition and hydration was established at home by oral intake alone.

After discharge, medical interviews including weight changes, physical examinations, imaging, and laboratory studies were routinely performed. Additional treatments such as chemotherapy and esophageal dilation of benign anastomotic strictures were provided as needed.

### Outcome Measures

#### Primary Outcome Measures

Skeletal muscle mass was measured using CT images taken 6 months after esophagectomy for regular follow-up purposes. Cross-sectional images at the level of the third lumbar vertebra were selected, and skeletal muscle was identified using Hounsfield unit (HU) thresholds between − 29 and + 150, according to previous studies [25, 26]. Image analysis was performed on a GE Healthcare AW Server version 2.0 (GE Healthcare). Skeletal muscle mass was reported as SMI (cm^2^/m^2^). One of the authors, blinded to participant characteristics and interventions, performed the measurements.

The diagnosis of sarcopenia was made using the cut-off criteria of SMI < 52.4 cm^2^/m^2^ for male and SMI < 38.5 cm^2^/m^2^ for female patients [25]. Skeletal muscle quality was reported as mean muscle attenuation (HU) for the entire muscle area at the third lumbar vertebra [26].

#### Secondary Outcome Measures

The following data were obtained for univariate and multivariate analyses:

- Participant characteristics consisted of age, sex, past medical history, height, and weight. Body mass index (BMI) was assessed from the preoperative period to the time of discharge and 6 months after surgery. Past medical history consisted of cerebrovascular disease, neuropsychiatric disease, head and neck surgery, and pneumonia, which could result in dysphagia and malnutrition. Vital capacity (%VC) and forced expiratory volume in 1 s (FEV1%) were measured as indicators of preoperative pulmonary function.
- Tumor characteristics consisted of pathological tumor stage and histology, categorized as squamous cell carcinoma or adenocarcinoma. The tumor stage was classified according to the tumor-node-metastasis (TNM) classification, 7th edition [27]. Neoadjuvant therapy consisted of chemotherapy, radiation therapy, or a combination of the two.
- Surgical factors consisted of: surgical procedures, categorized into thoracotomy versus video-assisted thoracoscopic surgery (VATS) and laparotomy versus hand-assisted laparoscopic surgery (HALS); lymph node dissection (three-field lymphadenectomy); operation time (minutes); and blood loss (mL).
- Postoperative complications included reoperation, pneumonia confirmed by chest X-ray or CT, anastomotic leakage, chylothorax, intra-abdominal abscess, empyema, and sepsis. Oral intake-related complications were defined as aspiration pneumonia that required discontinuation of oral feeding.
- Postoperative muscle strength was evaluated using handgrip strength on POD 8, measured according to the protocol recommended by the Asian Working Group for Sarcopenia (AWGS) [3].
- Nutritional status was assessed using albumin and the prognostic nutritional index (PNI) [28] from the preoperative period to 6 months after surgery.
- The inflammatory reaction was assessed using C-reactive protein (CRP) from the preoperative period to 6 months after surgery.
- Postoperative swallowing status was assessed using the food intake level scale (FILS) [29] until the time of discharge. FILS is one of the assessment tools for dysphagia in sarcopenic patients [30]. Based on the initial swallowing assessment using VFSS/FEES, patients with FILS ≤ 3 were classified as the non-oral intake group, whereas the patients with FILS ≥ 4 were classified as the oral intake group.
- A list of assessments is shown in Supplementary Table 1.

### Statistical Analysis

For interval scales, normality was checked using a histogram and the Shapiro–Wilk test. Normally distributed variables are expressed as means ± standard deviation, whereas non-normally distributed variables (e.g., POD, blood loss, operative time) are expressed as medians (interquartile range, IQR). Nominal and ordinal scales are expressed as numbers/percentages of patients. For comparisons between two groups, Student’s *t*-test or the Mann–Whitney *U* test was used for interval scales, and the chi-squared test or Fisher’s exact test was used for nominal and ordinal scales; split-plot analysis of variance and multiple comparisons with Bonferroni adjustment were used for time-dependent variables.

Multivariate logistic regression analysis was performed to identify perioperative risk factors during hospitalization that could be related to sarcopenia 6 months after esophagectomy, with the results presented as odds ratios (95% confidence interval, CI). The basic model adjusted for age and tumor stage was used, and all variables with *p* < 0.20 were included in the following multivariable model. Because of their clinical relevance, age and tumor stage were forcibly entered into the multivariable model. Multivariate analysis was performed by the backward elimination method (likelihood ratio; elimination criteria, *p* > 0.10). Statistical analysis was performed with SPSS version 25.0 (IBM). Values with *p* < 0.05 were considered significant.

## Results

### Participant Characteristics

**Fig. 1:**
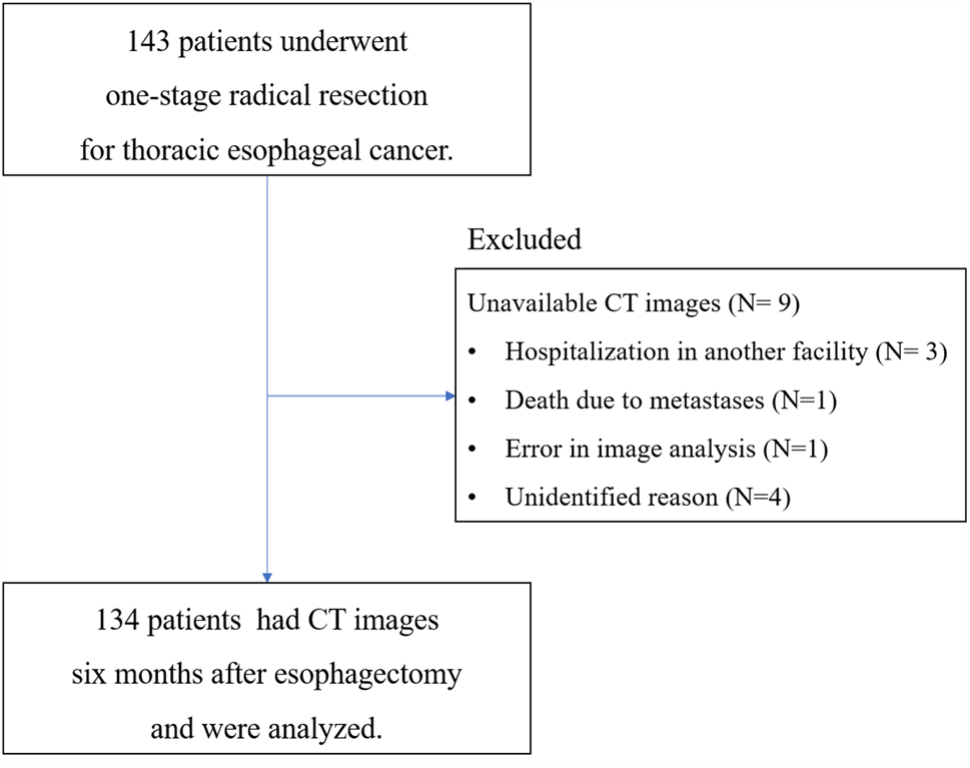
Flow diagram. The participants were sampled from patients with esophageal cancer who were referred to a university hospital for surgical treatment

Of the 143 patients identified, nine patients were excluded because CT images were not available (Fig. 1). Finally, the data of 134 patients were analyzed in this study. Significant sex differences were observed in preoperative height, weight, and BMI. In addition, postoperative handgrip strength, 6 month postoperative skeletal muscle mass, SMI, and mean attenuation were significantly lower in female than in male patients (Table 1). The prevalence of sarcopenia 6 months after esophagectomy was 35.1% (*n* = 47) overall, 41.4% (*n* = 46) in male patients, and 4.3% (*n* = 1) in female patients.

**Table 1:** Demographic characteristics of the participants

	Total (*n* = 134)	Men (*n* = 111)	Women (*n* = 23)	*P*-value
*Preoperative*				
Age	65.4 ± 8.2	65.5 ± 8.2	64.8 ± 8.3	0.703
Height (cm)	165.8 ± 7.8	168.4 ± 5.5	153.7 ± 5.2	< 0.001
Weight (kg)	59.2 ± 10.6	62.2 ± 8.7	44.9 ± 5.8	< 0.001
BMI (kg/m^2^)	21.4 ± 2.7	21.9 ± 2.6	19.0 ± 2.2	< 0.001
Past medical history	14 (10.4%)	11 (9.9%)	3 (13.0%)	0.444
%VC	108.5 ± 13.8	108.8 ± 14.0	107.3 ± 13.4	0.655
FEV1%	72.7 ± 9.3	72.2 ± 9.3	75.4 ± 9.1	0.147
Neoadjuvant therapy	82 (61.2%)	68 (61.3%)	14 (60.9%)	0.972
*Surgical resection*				
Tumor stage (0–2/3–4)	98/36	82/29	16/7	0.326
Histology (SCC/AC)	127/7	104/7	23/0	0.259
VATS/thoracotomy	116/18	96/15	20/3	0.628
HALS/laparotomy	108/26	87/24	21/2	0.125
Three-field lymphadenectomy	121 (90.3%)	98 (88.2%)	23 (100%)	0.076
Operation time (min)	485 (450–576)	486 (450–516)	455 (427–503)	0.139
Blood loss (mL)	100 (50–182)	110 (70–187)	60 (15–105)	0.004
*Postoperative*				
Handgrip strength (kg)	29.8 ± 8.6	32.2 ± 7.2	18.2 ± 3.9	< 0.001
Postoperative complications	69 (51.5%)	58 (52.3%)	11 (47.8%)	0.699
Chemotherapy within 6 months after esophagectomy	21 (15.7%)	19 (17.1%)	2 (8.7%)	0.252
Postoperative CT images (POD)	181 (161–197)	181 (162–194)	190 (148–212)	0.237
Skeletal muscle mass (cm^2^)	144.8 ± 29.8	153.5 ± 24.6	102.8 ± 10.8	< 0.001
SMI (cm^2^/m^2^)	52.4 ± 8.4	54.1 ± 7.9	43.6 ± 4.05	< 0.001
MA (HU)	40.2 ± 5.1	40.7 ± 5.2	37.9 ± 4.0	0.015

Data are expressed as mean ± SD, median (interquartile range), or number of patients (percentages). Differences between two groups are analyzed using Student’s t-test, Mann–Whitney *U* test, chi-square test, and Fisher’s exact test

*BMI* body mass index, *VC* vital capacity, *FEV1* forced expiratory volume in 1 s, *SCC* squamous cell carcinoma, *AC* adenocarcinoma, *VATS* video-assisted thoracoscopic esophagectomy, *HALS* hand-assisted laparoscopic surgery, *POD* postoperative day, *SMI* skeletal mass index, *MA* mean attenuation

### Swallowing Function and Oral Intake Status

The initial swallowing assessment using VFSS/FEES was performed on POD 12 (IQR, 8–14), and 46 patients (34.3%) were judged to be unable to start oral intake (FILS ≤ 3). The rate of vocal fold immobility on FEES and penetration/aspiration with thickened liquids on VFSS was significantly higher in the non-oral intake (NOI) group than in the oral intake (OI) group (97.8% vs. 27.3%, *p* < 0.001). The majority (85.7%) of patients who aspirated thickened liquids showed silent aspiration without a cough reflex, that is, categorized into PAS 8. The time to start oral intake, the time to finish diet modification (i.e., acquisition of regular food), and the date of discharge were POD 13 (IQR 9–18), 20 (IQR 15–29), and 26 (IQR 21–40), respectively, significantly longer in the NOI group than in the OI group (*p* < 0.001). On the other hand, the incidence of oral intake-related complication (i.e., aspiration pneumonia) was not significantly different between the NOI and OI groups from the initiation of oral intake until the time of discharge (10.9% vs. 5.7%, *p* = 0.226). For the patients unable to start oral intake on the initial swallowing assessment, the majority started oral intake on POD 17 (IQR 14–31), although a small number continued to receive tube feeding alone (FILS ≤ 3, 8.7% at discharge; 4.4% after 6 months).

At the time of discharge, a jejunostomy was placed in all participants but 30.6% (*n* = 41) actually received tube feeding with or without combined oral intake (FILS ≤ 6). In the NOI group on the initial swallowing assessment, a significantly higher proportion of patients received tube feeding at discharge than in the OI group (41.3% vs. 25.0%, *p* = 0.014). The jejunostomy status 6 months after surgery was not recorded for 10 patients (missing rate, 7.5%). Of 124 patients with records, 13.7% (*n* = 17) continued jejunostomy placement 6 months after esophagectomy; for the remainder, the jejunostomy was removed on POD 48 (IQR 38–73). The rate of jejunostomy placement 6 months after surgery was not significantly different between patients with and without sarcopenia (20.0% vs. 10.1%, *p* = 0.124). Of the 17 patients with continued jejunostomy placement 6 months after surgery, actual use of tube feeding was identified in 11 patients, while it was not documented for the remaining patients. Due to the incompleteness of the data, it was decided to exclude the use of tube feeding 6 months postoperatively in the statistical analysis.

### Body Composition, Nutritional Status, and Inflammatory Response

**Fig. 2:**
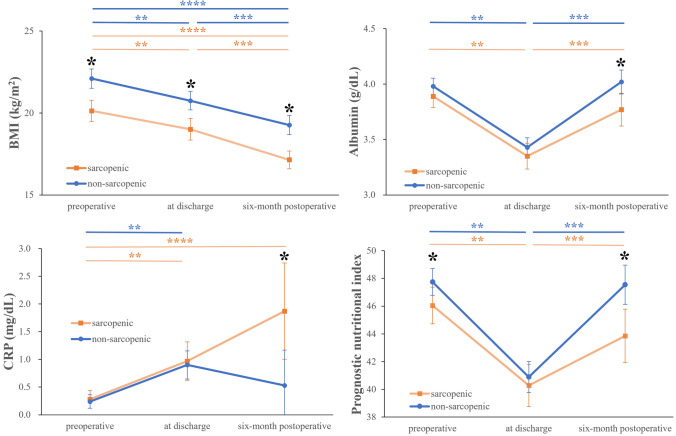
Changes in nutritional states from preoperatively to 6 months after surgery. Data are expressed as means ± 2SE. Differences between two groups and among three time points are examined with split-plot analysis and Bonferroni adjustment for multiple comparisons. A single asterisk (*) indicates statistically significant differences (*p* < .05) for postoperative sarcopenic group vs. non-sarcopenic group; double asterisks (**) for preoperatively vs. at discharge; triple asterisks (***) for at discharge vs. six-month postoperatively; and quadruple asterisks (****) for preoperatively vs. 6-month postoperatively. BMI body mass index, CRP C-reactive protein, SE standard error

The rate of missing data for body weight and thus BMI 6 months after esophagectomy was 47.8%; therefore, it was decided to exclude this from the multivariate analysis. Of the 70 patients whose medical records were available, the mean body weight 6 months postoperatively was 51.2 ± 8.6 kg, and the percentage of patients with weight loss of at least 10% between before and 6 months after esophagectomy was 68.6%. BMI as a body composition indicator decreased progressively from the preoperative period to 6 months after surgery in both the postoperative sarcopenic and non-sarcopenic groups (Fig. 2). At any point in time, the postoperative sarcopenic group had significantly lower BMI than the postoperative non-sarcopenic group. Albumin and PNI as nutritional indicators decreased significantly from the preoperative period to the time of discharge, but they subsequently improved to the preoperative levels within 6 months postoperatively in both sarcopenic and non-sarcopenic groups. The sarcopenic group showed significantly lower PNI than the non-sarcopenic group before and 6 months after surgery. The sarcopenic group also showed significantly lower albumin 6 months after surgery, and average values never reached the normal levels at any point in time (3.89 ± 0.10 on admission, 3.35 ± 0.12 at discharge, 3.77 ± 0.15 6 months after surgery). CRP as an inflammatory response indicator increased from the preoperative period to the time of discharge in both groups and then continued at high levels in the sarcopenic group, whereas it decreased to the preoperative level 6 months after surgery in the non-sarcopenic group. The sarcopenic group showed significantly higher CRP levels than the non-sarcopenic group 6 months after surgery.

### Multivariate Logistic Regression Analysis

The missing rate for each item was as follows: preoperative pulmonary function test, 0.75%; postoperative handgrip strength, 0.75%; BMI at discharge, 4.5%. Missing data for independent variables were few and therefore excluded from the multivariate analysis. Since only one female patient was diagnosed with sarcopenia, a subsequent multivariate analysis included only male patients (*n* = 111).

**Fig. 3:**
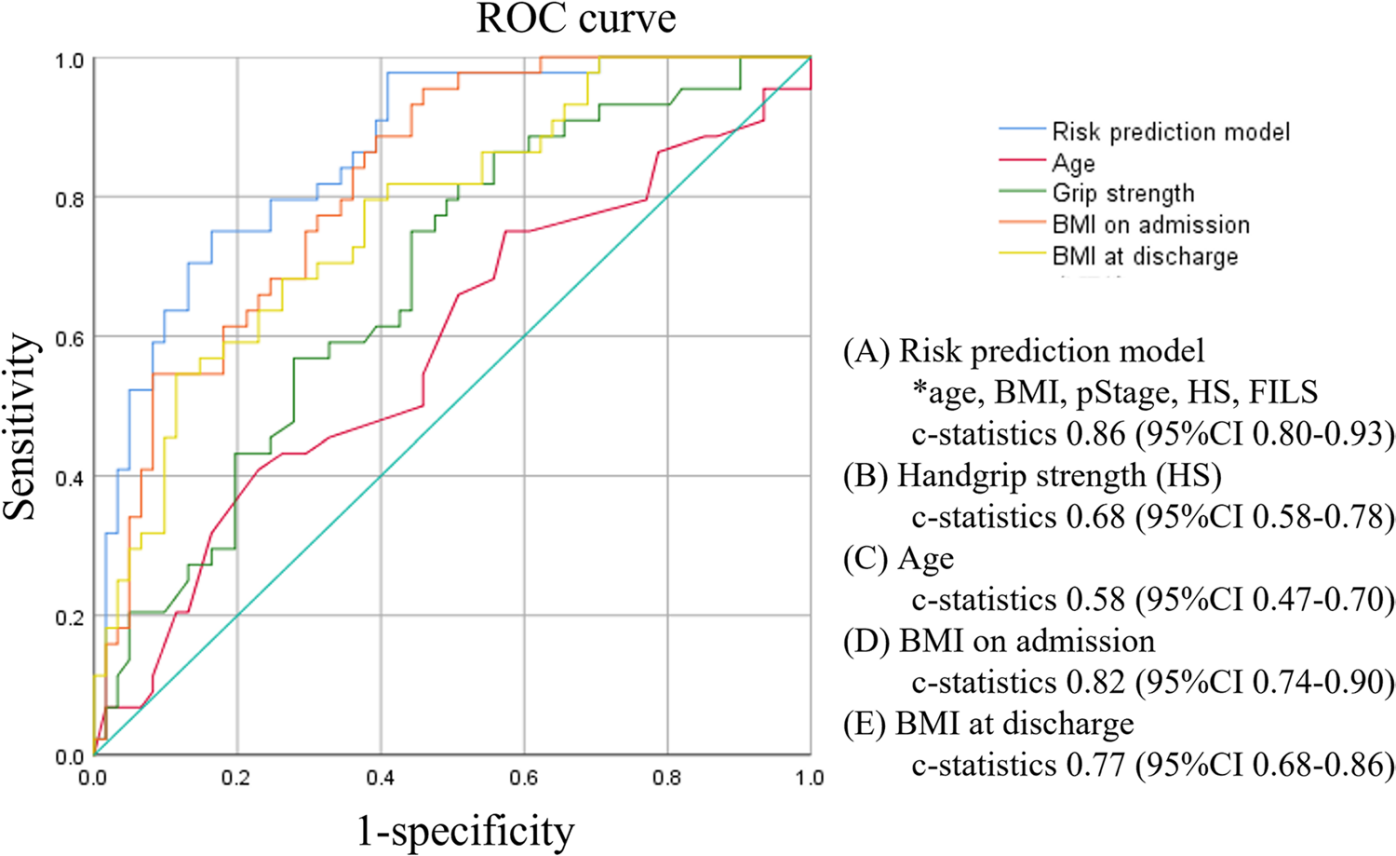
Comparisons of c-statistics among the risk prediction models using each clinical parameter. The risk prediction model includes age, preoperative BMI, pathological tumor stage, postoperative handgrip strength, and use of tube feeding at discharge

Multivariate analysis using the basic model showed that preoperative BMI and postoperative handgrip strength had a significantly negative association, whereas use of tube feeding at discharge (versus oral intake alone) had a significantly positive association with sarcopenia 6 months after esophagectomy (Table 2). Age, preoperative BMI, preoperative PNI, preoperative %VC, tumor stage, postoperative handgrip strength, inability to start oral intake (FILS ≤ 3) on the initial swallowing assessment, and use of tube feeding (FILS ≤ 6) at discharge were included in the multivariate model. Preoperative BMI, postoperative handgrip strength, and use of tube feeding at discharge were independent risk factors for sarcopenia 6 months after esophagectomy (Table 2). To assess the ability to predict outcomes, c-statistics were examined. The goodness of fit of the prediction model was very good, with high c-statistics (0.86; 95% CI 0.80–0.93) (Fig. 3).

**Table 2:** Multivariate logistic regression analysis of risk factors for sarcopenia postoperatively in male patients with esophageal cancer

Factors	Objective variables	Reference	Basic model*		Multivariable model^#^	
			Odds ratio [95%CI]	*P*-value	Odds ratio [95%CI]	*P*-value
*Preoperative*						
Age			1.04 [0.99, 1.09]	0.142	0.97 [0.90, 1.04]	0.374
BMI			0.55 [0.43, 0.71]	< 0.001	0.56 [0.43, 0.74]	< 0.001
PNI			0.92 [0.84, 1.01]	0.088		
Past medical history	Present	Absent	1.96 [0.54, 7.09]	0.303		
%VC			0.97 [0.94, 1.00]	0.055		
FEV1%			0.99 [0.95, 1.04]	0.763		
Neoadjuvant therapy	Present	Absent	1.64 [0.70, 3.86]	0.259		
*Surgical resection*						
Tumor stage	3–4	0–2	2.26 [0.96, 5.36]	0.064	2.51 [0.83, 7.65]	0.105
Histology	SCC	AC	1.74 [0.29, 10.34]	0.545		
Surgical procedures	VATS	Thoracotomy	0.66 [0.21, 2.06]	0.472		
	HALS	Laparotomy	0.82 [0.32, 2.13]	0.688		
Three-field lymphadenectomy	Present	Absent	1.28 [0.37, 4.45]	0.697		
*Postoperative*						
Handgrip strength			0.88 [0.82, 0.96]	0.003	0.90 [0.82, 0.98]	0.018
Postoperative complications	Present	Absent	1.60 [0.73, 3.50]	0.241		
Oral intake ability on initial swallowing assessment	Non-oral intake (FILS ≤ 3)	Oral intake (FILS ≥ 4)	1.93 [0.85, 4.40]	0.118		
Use of tube feeding at discharge	Present (FILS ≤ 6)	Absent (FILS ≥ 7)	2.47 [1.03, 5.93]	0.043	3.23 [1.05, 9.90]	0.041

*BMI* body mass index, *PNI* prognostic nutritional index, *VC* vital capacity, *FEV1* forced expiratory volume in 1 s, *SCC* squamous cell carcinoma, *AC* adenocarcinoma, *VATS* video-assisted thoracoscopic esophagectomy, *HALS* hand-assisted laparoscopic surgery, *FILS* food intake level scale

*The basic model includes age and pathological tumor stage

^#^The multivariate model (backward elimination method) includes age, preoperative BMI, preoperative PNI, preoperative %VC, tumor stage, postoperative handgrip strength, oral intake ability on initial swallowing assessment, and use of tube feeding at discharge. Age and tumor stage are forced into the multivariate model

## Discussion

The present results showed that significant numbers of male patients developed sarcopenia 6 months after esophagectomy, and use of tube feeding at discharge was an independent predictor, in addition to postoperative handgrip strength and preoperative BMI. To the best of our knowledge, this is the first study to demonstrate that the need for alternative nutrition is related to postoperative sarcopenia in patients with esophageal cancer.

### Prevalence of Sarcopenia Six Months After Esophagectomy

The SMI has no standard cut-off values. In the present study, the cut-off that is most commonly used in studies for patients with esophageal cancer, including Asian populations, was selected [31]. A previous study using the same cut-off reported that the prevalence of sarcopenia was 6.9% before neoadjuvant therapy, 21.1% after neoadjuvant therapy, and 34.7% 1 year after esophagectomy [12]. The present study showed that the prevalence of sarcopenia 6 months after esophagectomy was 35.1%, which is consistent with the previous study. On the other hand, the present results showed a large difference in prevalence between male and female patients. A previous study of Asian patients with esophageal cancer using the same cut-off also reported sex differences in the prevalence of sarcopenia [32]. The cut-off values used in the present study were lower in female than in male patients. Higher cut-offs of SMI are shown to significantly increase the prevalence of sarcopenia [30], which might have affected the results of the present study.

### Dysphagia, Oral Intake Status, and Nutritional Status During the Perioperative Period and Six Months After Surgery

This study showed that 34.3% of patients were judged to be unable to start oral intake at 1 week after esophagectomy, which is consistent with a previous study [33]. These patients were significantly more likely to receive tube feeding at discharge, suggesting a good indication for prioritized nutrition education. With low rates of jejunostomy placement 6 months after surgery, the majority of participants took nutrients by oral intake alone. However, the percentage of patients who experienced weight loss of at least 10% between before and 6 months after esophagectomy accounted for 68.6%, showing higher rates than those of a previous study [34]. In addition, other nutritional indicators 6 months after surgery were lower than those in a previous study [35], especially in the sarcopenic group, thus suggesting the importance of continuous nutritional assessment and supplementation after discharge [34].

The present study showed that 3.7% (*n* = 5) of the patients were unable to start oral intake at discharge, which is consistent with a previous report [36]. The present results also showed that 1.5% (*n* = 2) of the patients continued to receive tube feeding alone 6 months after esophagectomy. Pharyngeal dysphagia and recurrent nerve paralysis were reported to persist 6 months after surgery in some patients with esophageal cancer [37, 38]. It is therefore necessary to assess swallowing function and nutritional status sequentially after discharge and provide appropriate swallowing training accordingly.

### Perioperative Risk Factors for Sarcopenia Six Months After Esophagectomy

Factors that impact sarcopenia in patients with cancer include aging, inflammation, physical activity, and nutritional status [2]. This study showed that need for alternative nutrition at discharge, as well as postoperative handgrip strength and preoperative BMI, was an independent predictor of sarcopenia 6 months after surgery in patients with esophageal cancer. Handgrip strength is one of the criteria to detect sarcopenia [6, 7]. Muscle weakness was reported to precede skeletal muscle loss [5], and, therefore, it is reasonable that postoperative handgrip strength was a predictive factor for sarcopenia 6 months after surgery.

The present results also showed that lower preoperative BMI was significantly associated with sarcopenia 6 months after surgery. A similar trend was observed in a previous study in which few patients with sarcopenic obesity were included [12]. Few obese patients were included in the present study, and body weight might have reflected skeletal muscle mass. In contrast, a previous study including many obese patients reported that higher preoperative BMI was associated with postoperative weight loss [18]. These findings suggest that not only body weight, but also body composition including muscle and fat should be assessed preoperatively.

The use of tube feeding at discharge was a significant risk factor for sarcopenia 6 months after esophagectomy compared with oral intake alone. One possible explanation was insufficient energy intake. Persistent dysphagia made it difficult to secure sufficient energy by oral intake alone; thus, energy intake was made dependent on tube feeding, and it is possible that the caloric requirement for increased activity after discharge might not be sufficiently secured. In addition, chronic inflammation of the trachea or bronchus due to subclinical aspiration further increases the caloric requirement, which was partially supported by the finding that 6 month postoperative CRP was significantly higher in the sarcopenic group than in the non-sarcopenic group. Another potential explanation was differences in nutritional intake routes. Okuda et al. [35] reported that poor oral intake was a contributor to negative outcomes even after calorie intake was adjusted and suggested the possibility that the cephalic phase response, which prepares the gastrointestinal tract for digestion and absorption of food by promoting physiological changes before food intake, affected the outcomes. Since the present results do not preclude the possibility, it might be better to consider combined use of tube feeding at discharge as an overall risk factor.

The present study had several limitations. First, this was a cross-sectional study, and examination of causal relationships was not possible. Second, caution should be paid when applying these results to female patients, although the prevalence of esophageal cancer was innately low in female patients and would not interfere with the generalization of the present results. Third, the prevalence of sarcopenia should be interpreted with caution in this single-center study. Although postoperative management of nutritional status was uniformly provided in our hospital, different hospitals might offer different postoperative care and resources, which might change the calories administered and thus affect the prevalence of sarcopenia. To address this, the calories administered should be noted in future studies, which would facilitate comparison between facilities. Finally, the availability of some preoperative and postoperative data was limited due to the retrospective nature of the present study. In this study, factors affecting postoperative sarcopenia were retrospectively investigated, and analysis of preoperative sarcopenia was outside the scope of this study. The findings suggest that preoperative sarcopenia should be investigated in future studies for several reasons: Preoperative BMI was identified as a factor associated with postoperative sarcopenia in this study. Although BMI might have reflected skeletal muscle mass in this cohort, a prospective observational study assessing preoperative sarcopenia would clarify the causal relationship. In addition, use of tube feeding at discharge was also identified as a factor associated with postoperative sarcopenia, and future studies assessing both preoperative and postoperative sarcopenia would provide new insights into the contribution of tube feeding to persistent and new-onset sarcopenia, respectively. Similarly, the medical records were carefully reviewed to explore the nutritional status 6 months after esophagectomy, but it was difficult to obtain accurate information on body weight and use of tube feeding for all patients. In addition, gastrointestinal symptoms such as anorexia were not assessed in this study, which might affect the use of tube feeding. These issues should be resolved in future prospective observational studies.

## Conclusion

In patients who underwent esophagectomy, use of tube feeding at discharge was identified as a significant risk factor for postoperative sarcopenia. Identifying high-risk groups based on preoperative BMI, postoperative handgrip strength, and use of tube feeding at discharge might allow early detection of malnutrition and provision of appropriate nutritional support and exercise.

## Supplementary Information

Below is the link to the electronic supplementary material.

Supplementary file1 (DOCX 33 kb)

## Data Availability

The datasets during and/or analyzed during the present study available from the corresponding author on reasonable request.
