# Correction to: Effect of Postoperative Oral Intake Status on Sarcopenia Six Months After Esophageal Cancer Surgery

**DOI:** 10.1007/s00455-022-10540-3

**Published:** 2022-12-21

**Authors:** Nanako Hijikata, Aiko Ishikawa, Satoru Matsuda, Michiyuki Kawakami, Kaori Muraoka, Makiko Ando, Shuhei Mayanagi, Tomoyuki Irino, Hirofumi Kawakubo, Yuko Kitagawa, Tetsuya Tsuji

**Affiliations:** 1 Department of Rehabilitation Medicine, Keio University School of Medicine, 35 Shinanomachi, Shinjuku‑ku, Tokyo, 160‑8582 Japan; 2 Department of Rehabilitation Medicine, National Cancer Center Hospital East, Kashiwa, Chiba Japan; 3 Department of Surgery, Keio University School of Medicine, Tokyo, Japan; 4 Department of Rehabilitation Medicine, Kitasato University Kitasato Institute Hospital, Tokyo, Japan; 5 Department of Rehabilitation Medicine, Keio University Hospital, Tokyo, Japan; 6 Division of Esophageal Surgery, Shizuoka Cancer Center Hospital, Shizuoka, Japan

## Correction to: Dysphagia 10.1007/s00455-022-10471-z

The original version of this article unfortunately contained a mistake. The footnotes in Table 2 were incorrect since sentences were in the wrong order during the publication process. The correct version of Table 2 is given below.

**Table 2:** Multivariate logistic regression analysis of risk factors for sarcopenia postoperatively in male patients with esophageal cancer

Factors	Objective variables	Reference	Basic model*		Multivariable model#	
			Odds ratio [95%CI]	*P*-value	Odds ratio [95%CI]	*P*-value
*Preoperative*						
Age			1.04 [0.99, 1.09]	0.142	0.97 [0.90, 1.04]	0.374
BMI			0.55 [0.43, 0.71]	< 0.001	0.56 [0.43, 0.74]	< 0.001
PNI			0.92 [0.84, 1.01]	0.088		
Past medical history	Present	Absent	1.96 [0.54, 7.09]	0.303		
%VC			0.97 [0.94, 1.00]	0.055		
FEV1%			0.99 [0.95, 1.04]	0.763		
Neoadjuvant therapy	Present	Absent	1.64 [0.70, 3.86]	0.259		
*Surgical resection*						
Tumor stage	3–4	0–2	2.26 [0.96, 5.36]	0.064	2.51 [0.83, 7.65]	0.105
Histology	SCC	AC	1.74 [0.29, 10.34]	0.545		
Surgical procedures	VATS	Thoracotomy	0.66 [0.21, 2.06]	0.472		
	HALS	Laparotomy	0.82 [0.32, 2.13]	0.688		
Three-field lymphadenectomy	Present	Absent	1.28 [0.37, 4.45]	0.697		
*Postoperative*						
Handgrip strength			0.88 [0.82, 0.96]	0.003	0.90 [0.82, 0.98]	0.018
Postoperative complications	Present	Absent	1.60 [0.73, 3.50]	0.241		
Oral intake ability on initial swallowing assessment	Non-oral intake (FILS ≤ 3)	Oral intake (FILS ≥ 4)	1.93 [0.85, 4.40]	0.118		
Use of tube feeding at discharge	Present (FILS ≤ 6)	Absent (FILS ≥ 7)	2.47 [1.03, 5.93]	0.043	3.23 [1.05, 9.90]	0.041

*BMI* body mass index, *PNI* prognostic nutritional index, *VC* vital capacity, *FEV1* forced expiratory volume in 1 s, *SCC* squamous cell carcinoma, *AC* adenocarcinoma, *VATS* video-assisted thoracoscopic esophagectomy, *HALS* hand-assisted laparoscopic surgery, *FILS* food intake level scale

^*^The basic model includes age and pathological tumor stage

#The multivariate model (backward elimination method) includes age, preoperative BMI, preoperative PNI, preoperative %VC, tumor stage, postoperative handgrip strength, oral intake ability on initial swallowing assessment, and use of tube feeding at discharge. Age and tumor stage are forced into the multivariate model

The original article has been corrected.

