# Supplementary material for: Effect of Postoperative Oral Intake Status on Sarcopenia Six Months After Esophageal Cancer Surgery
Source: Dysphagia. 2022 Jun 18;38(1):340–50. doi: 10.1007/s00455-022-10471-z (PMC9873756; doi:10.1007/s00455-022-10471-z)
Supplement: Supplementary file 1 — Supplementary file1 (DOCX 33 kb) [file 455_2022_10471_MOESM1_ESM.docx]

# Supplementary Table 1. List of outcome measures

|  | Preoperative (on admission) | Surgical resection | Postoperative  until the date of discharge | Six months postoperative |
| --- | --- | --- | --- | --- |
| Patient characteristics | age, gender, past medical history, height, weight, BMI,  %VC, FEV1% |  | weight, BMI (at discharge) | weight, BMI |
| Tumor characteristics | neoadjuvant therapy | tumor stage, histology |  | chemotherapy within six months after esophagectomy |
| Surgical characteristics |  | surgical procedures, lymph node dissection, operation time, blood loss | postoperative complications |  |
| Muscle strength |  |  | handgrip strength (POD 8) |  |
| Muscle quantity and quality |  |  |  | skeletal muscle mass, SMI, mean attenuation |
| Nutrition/ Inflammation | Alb, CRP, PNI |  | Alb, CRP, PNI (at discharge) | Alb, CRP, PNI |
| Swallowing and oral intake status |  |  | VFSS/FEES (POD 8)  FILS (at discharge) | jejunostomy status |

BMI, body mass index; VC, Vital Capacity; FEV1, forced expiratory volume in 1 s; POD, postoperative day; SMI, skeletal mass index; Alb, Albumin; CRP, C-reactive protein; PNI, prognostic nutritional index; VFSS, videofluoroscopic swallow study; FEES, fiberoptic endoscopic evaluation of swallowing; FILS, food intake level scale.
